# Supplementary material for: Investigating the inflammation marker neutrophil-to-lymphocyte ratio in Danish blood donors with restless legs syndrome
Source: PLoS One. 2021 Nov 12;16(11):e0259681. doi: 10.1371/journal.pone.0259681 (PMC8589184; doi:10.1371/journal.pone.0259681)
Supplement: S1 Fig — (PDF) [file pone.0259681.s001.pdf]

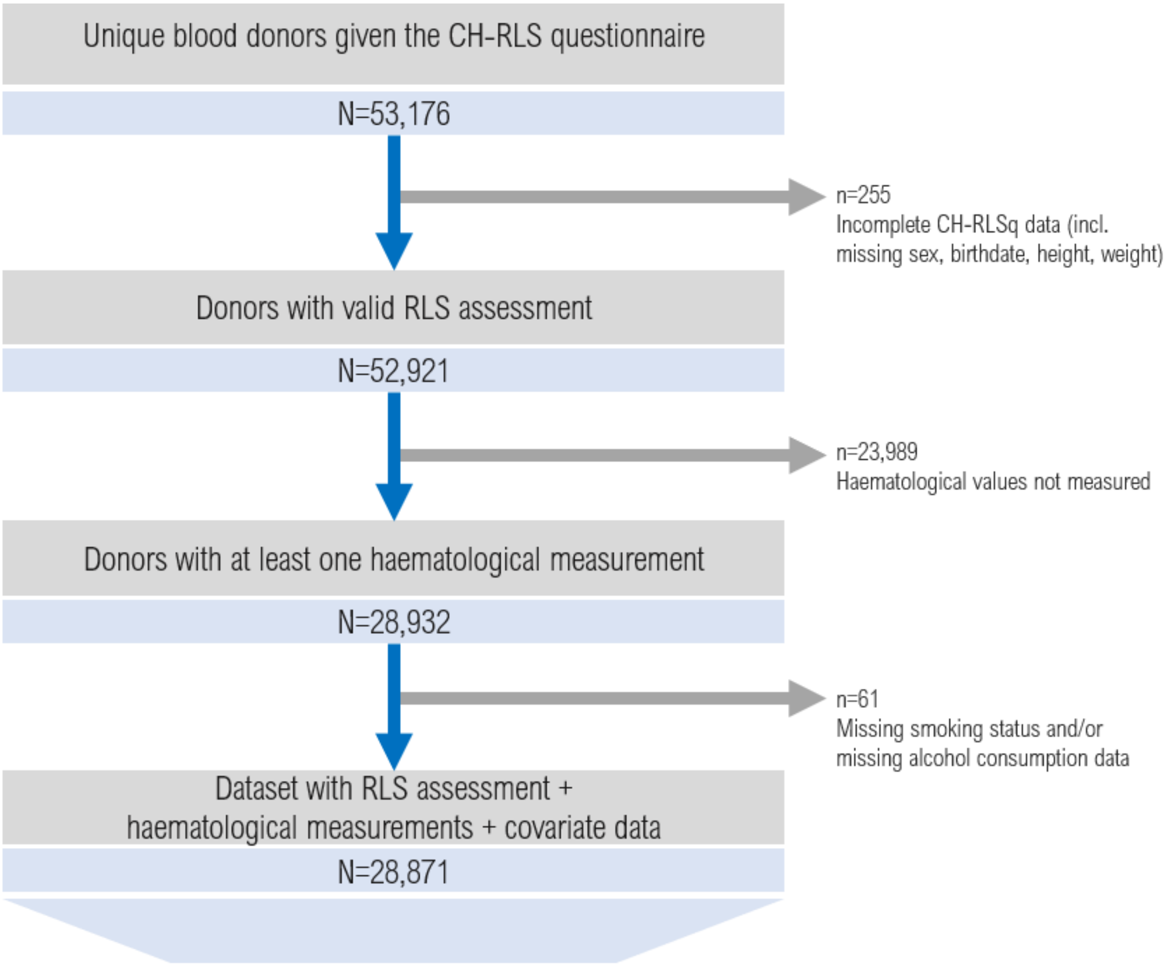

Marker	Measured in		Total N
	The Central Denmark Region	The Capital Region of Denmark	
WBC	N=14,356	N=14,515	28,871
MCV	N=14,356	N=14,513	28,869
MCHC	N=14,356	N=14,513	28,869
Platelets	N=14,353	N=14,513	28,866
PDW	N=13,147	N=14,477	27,624
PLCR	N=13,147	N=14,477	27,624
Neutrophils	N=13,040	N=15	13,055
Lymphocytes	N=13,040	N=15	13,055

S1 Fig: **Flowchart of the inclusion process**. CH-RLSq = Cambridge Hopkins RLS questionnaire. WBC = white blood cells, MCV = mean corpuscular volume, MCHC = mean corpuscular haemoglobin concentration. PDW = platelet distribution width, PLCR = platelet large cell ratio.
