# Supplementary material for: Investigating the inflammation marker neutrophil-to-lymphocyte ratio in Danish blood donors with restless legs syndrome
Source: PLoS One. 2021 Nov 12;16(11):e0259681. doi: 10.1371/journal.pone.0259681 (PMC8589184; doi:10.1371/journal.pone.0259681)
Supplement: S2 Fig — (PDF) [file pone.0259681.s002.pdf]

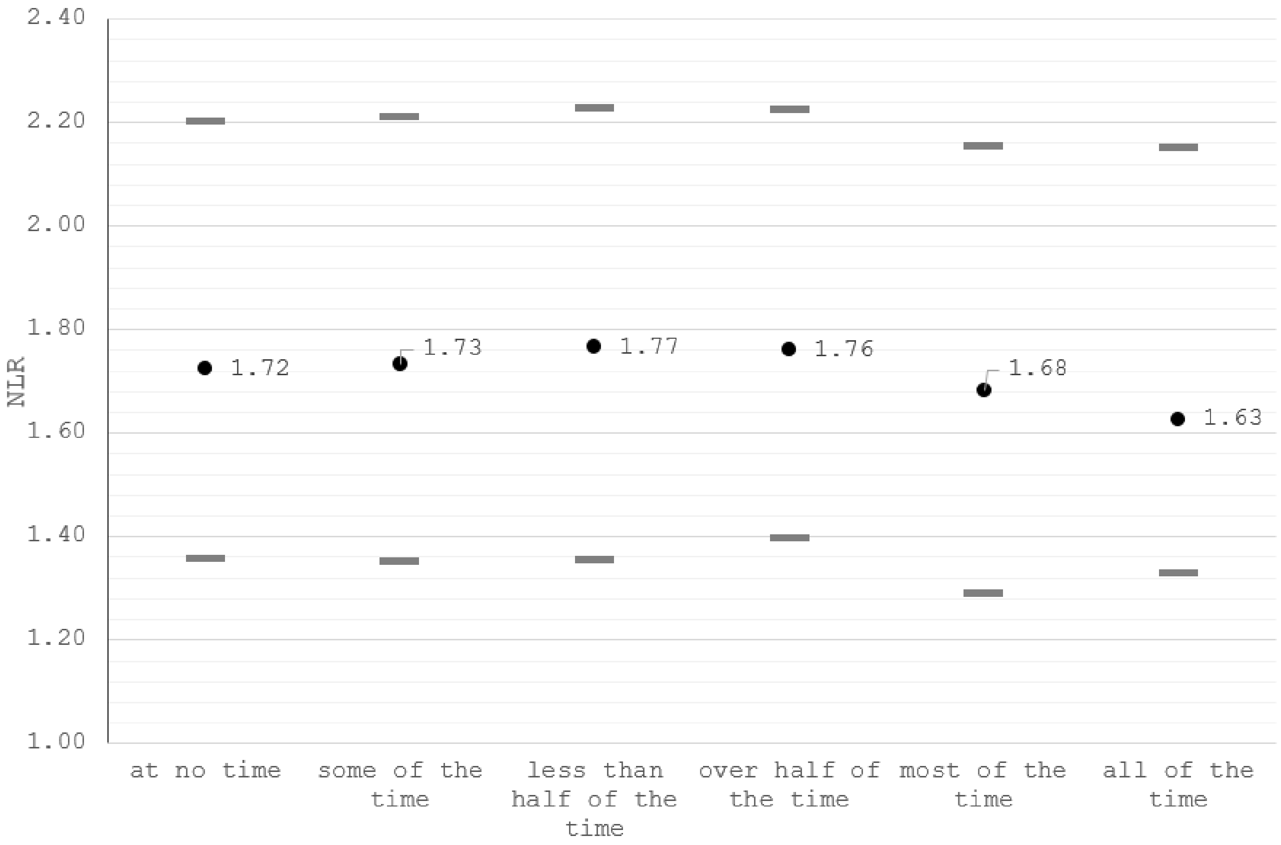

S2 Fig. Median NLR and Difficulty Sleeping. Blood donors' answers to the question "Over the last 2 weeks, how much of the time have you had trouble sleeping at night?" and the corresponding median NLR in each group in the DBDS NLR-RLS dataset, excluding 39 who did not answer the question (N=13,016). Grey stripes denote the interquartile ranges.
