# Supplementary material for: Investigating the inflammation marker neutrophil-to-lymphocyte ratio in Danish blood donors with restless legs syndrome
Source: PLoS One. 2021 Nov 12;16(11):e0259681. doi: 10.1371/journal.pone.0259681 (PMC8589184; doi:10.1371/journal.pone.0259681)
Supplement: S4 Fig — (PDF) [file pone.0259681.s004.pdf]

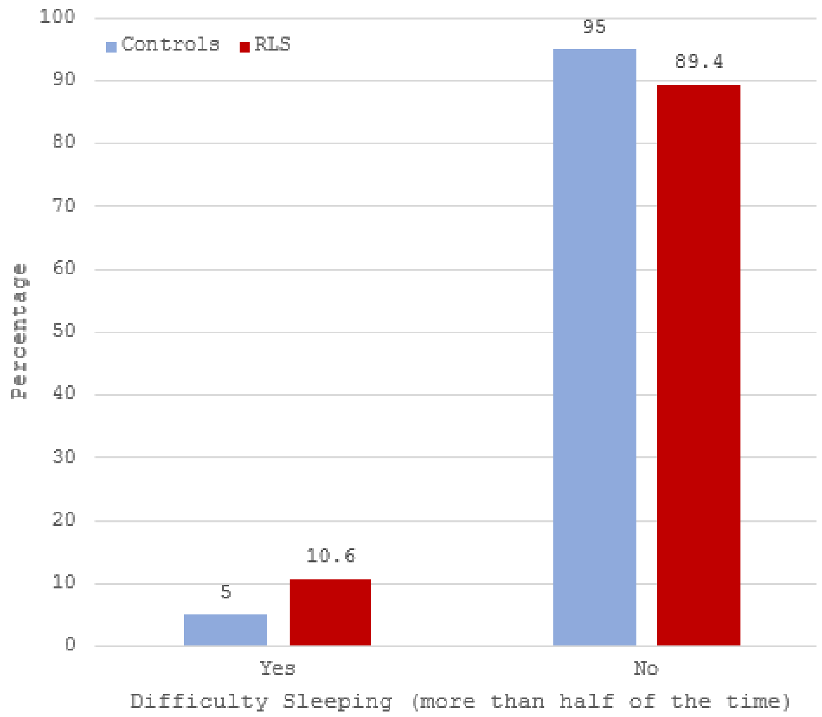

S4 Fig. Proportion of RLS cases and controls reporting that they have had difficulty sleeping (as binary variable) in the two weeks leading up to the donation in the DBDS NLR-RLS dataset, excluding 39 who did not answer the question (N=13,016).
