# Supplementary material for: Investigating the inflammation marker neutrophil-to-lymphocyte ratio in Danish blood donors with restless legs syndrome
Source: PLoS One. 2021 Nov 12;16(11):e0259681. doi: 10.1371/journal.pone.0259681 (PMC8589184; doi:10.1371/journal.pone.0259681)
Supplement: S5 Fig — (PDF) [file pone.0259681.s005.pdf]

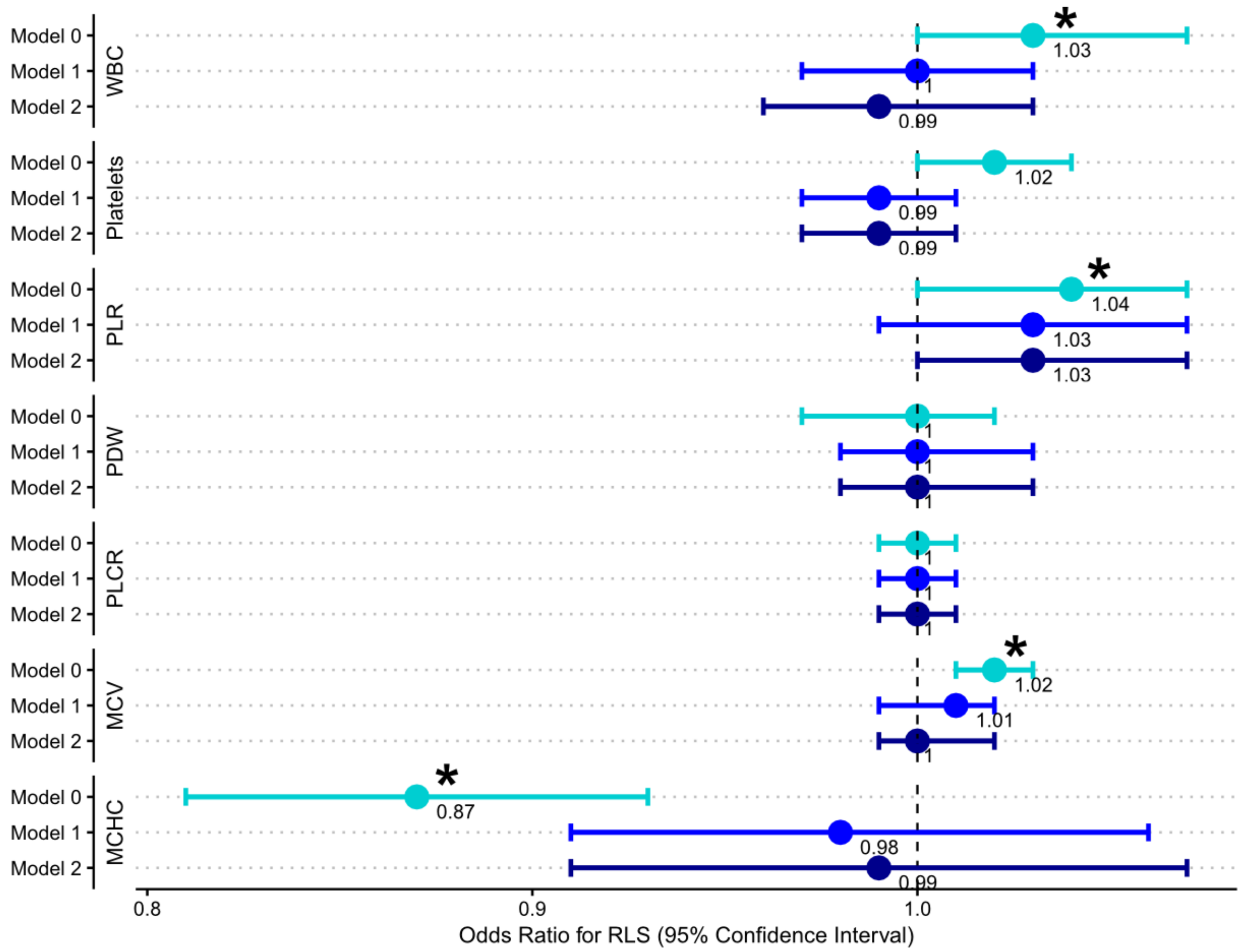

S5 Fig. Haematological markers as predictors of RLS. Results from the logistic regression models using seven haematological markers as the predictor variable and RLS status as the outcome in the DBDS cohort. Model 0 = Crude association. Model 1 = adjusting for sex and age. Model 2 = adjusting for sex, age, alcohol consumption, smoking, BMI and region of donation. Data are presented as odd ratios (OR) with 95% confidence intervals and an asterisk (*) denotes a P value < 0.05. OR for platelets and PLR are for a 20-unit increase. WBC = white blood cells, PLR = platelet-to-lymphocyte ratio, PDW = platelet distribution width, PLCR = platelet large cell ratio, MCV = mean corpuscular volume, MCHC = mean corpuscular haemoglobin concentration.
