# Supplementary material for: Investigating the inflammation marker neutrophil-to-lymphocyte ratio in Danish blood donors with restless legs syndrome
Source: PLoS One. 2021 Nov 12;16(11):e0259681. doi: 10.1371/journal.pone.0259681 (PMC8589184; doi:10.1371/journal.pone.0259681)
Supplement: S1 Table — (PDF) [file pone.0259681.s006.pdf]

S1 Table: Proportion of RLS cases and controls reporting that they have had difficulty sleeping (at least more than half of the time) in the two weeks leading up to the donation in the DBDS NLR-RLS dataset, excluding 39 who did not answer the question (N=13,016).

	**Controls**		**RLS Cases**		
	**N**	**%**	**N**	**%**	**P value**[a]
**Difficulty Sleeping**[b]					
Yes	621	5.0	70	10.6	**<0.001**
No	11,736	95.0	589	89.4	

[a]chi-square test.

[b]A donor is considered having "difficulty sleeping" when they answer either "more than half of the time", "most of the time" or "all of the time" to the question "Over the last two weeks, how much of the time have you had trouble sleeping at night?".
