# Supplementary material for: Investigating the inflammation marker neutrophil-to-lymphocyte ratio in Danish blood donors with restless legs syndrome
Source: PLoS One. 2021 Nov 12;16(11):e0259681. doi: 10.1371/journal.pone.0259681 (PMC8589184; doi:10.1371/journal.pone.0259681)
Supplement: S2 Table — (PDF) [file pone.0259681.s007.pdf]

S2 Table: **Descriptive statistics for RLS cases and controls for other haematological markers.** Demographic descriptive statistics for RLS cases and controls in the Danish Blood Donor Study with other haematological measurements available (N=28,871).

	**Controls N= 27,431**		**RLS Cases N= 1,440 (5.0%)**		
	**N**	**%**	**N**	**%**	**P value**[a]
**Demographic characteristics**					
**Sex**					
Male	14,011	51.1	550	38.2	**<0.001**
Female	13,420	48.9	890	61.8	
**Age**					
median (IQR**)**	37.6 (27.1-49.6)		39.7 (28.3-50.3)		**<0.001**
**BMI**					
median (IQR)	24.7 (22.5-27.2)		24.7 (22.6-27.8)		**0.044**
<18.5	171	0.6	8	0.6	0.374
18.5-25	14,862	54.2	760	52.8	
25-30	9,325	34.0	484	33.6	
30-35	2,285	8.3	142	9.9	
35-40	596	2.2	33	2.3	
>40	192	0.7	13	0.9	
**Smoking status**					
Non-smoker	23,935	87.3	1,226	85.1	**0.034**
<1 cigarette per day	1,286	4.7	71	4.9	
>1 cigarette per day	2,210	8.1	143	9.9	
**Alcohol consumption**					
Never/almost never	3,607	13.1	182	12.6	0.793
A couple of times a month	14,378	52.4	753	52.3	
A couple of times a week	8,355	30.5	441	30.6	
Daily/almost daily	1,091	4.0	64	4.4	
**Region Measured**					
Capital Region of Denmark	13,801	50.3	714	49.6	0.590
Region of Central Denmark	13,630	49.7	726	50.4	
**Haematological Measurements**					
**White Blood Cells** (N=28,871) 10^9/L, median (IQR)	6.26 (5.31-7.39)		6.38 (5.34-7.52)		0.050
**Platelets** (N=28,866) 10^9/L, median (IQR)	240 (207-277)		241 (209-280)		0.059
**Platelet-to-Lymphocyte Ratio (PLR)** (N=13,055) Median (IQR)	125.14 (103.33-152.40)		126.34 (100.00-159.80)		0.282
**Platelet Distribution Width** (N=27,624) fL, median (IQR)	13.6 (12.4-15.0)		13.5 (12.3-15.1)		0.627
**Platelet Large Cell Ratio** (N=27,624) %, median (IQR)	34.5 (29.6-39.7)		34.4 (29.6-40.0)		0.805
**Mean Corpuscular Volume** (N=28,869) fL, median (IQR)	89.8 (87.1-92.5)		90.0 (87.3-93.0)		**0.002**
**Mean Corpuscular Haemoglobin Concentration** (N=28,869) mmol/L, median (IQR)	20.6 (20.2-21.1)		20.6 (20.1-21.0)		**<0.001**

[a]For comparison of the two groups, chi-square test was used for categorical variables and Kruskal-Wallis rank test was used for continuous variables.
